# Prevalence and burden of primary headache disorders among a local community in Addis Ababa, Ethiopia

**DOI:** 10.1186/1129-2377-14-30

**Published:** 2013-03-28

**Authors:** Getahun Mengistu, Samson Alemayehu

**Affiliations:** 1 Department of Neurology, School of Medicine, Addis Ababa University, P.O. Box: 2380, Addis Ababa, Ethiopia

**Keywords:** Prevalence, Primary headaches, Burden, Addis Ababa, Ethiopia

## Abstract

**Background:**

Headache disorders are the most common complaints worldwide. Migraine, tension type and cluster headaches account for majority of primary headaches and improvise a substantial burden on the individual, family or society at large. There is a scanty data on the prevalence of primary headaches in sub-Saharan Africa in general and Ethiopia in particular. Moreover there is no population based urban study in Ethiopia. The purpose of this study is to determine the prevalence and burden of primary headaches in local community in Addis Ababa, Ethiopia.

**Methods:**

Cross-sectional sample survey was carried out in Addis Ketema sub city, Kebele 16/17/18 (local smallest administrative unit). Using systematic random sampling, data were collected by previously used headache questionnaire, over a period of 20 days.

**Results:**

The study subjects were 231 of which 51.5% were males and 48.5% were females. The overall one year prevalence of primary headache disorders was 21.6% and that for migraine was 10%, migraine without aura 6.5% migraine with aura was 2.6% and probable migraine was 0.9%. The prevalence of tension type of headache was found to be 10.4%, frequent episodic tension type headache was 8.2% followed by infrequent tension type headache of 2.2%. The prevalence of cluster headache was 1.3%. The burden of primary headache disorders in terms of missing working, school or social activities was 68.0%. This was 78.3% for migraineurs and 66.7% for tension type headache. Majority 92.0% of primary headache cases were not using health services and 66.0% did not use any drug or medications during the acute attacks and none were using preventive therapy.

**Conclusion:**

Prevalence and burden of primary headache disorders was substantial in this community. Health service utilization of the community for headache treatment was poor.

## Background

Headache is one among the most common medical complaints. Various forms of headache, properly called headache disorders, are among the most common disorders of the nervous system. They are pandemic and, in many cases, life-long conditions [1]. As many as 90% of all primary headaches including: migraine, tension type and cluster headache, fall under few categories. Recurrent headache disorders impose a substantial burden on headache sufferers, family and society [2]. Headache disorders are in the top ten-and possibly the top five-causes of disability worldwide [3].

Population-based studies in the world have mostly focused on migraine which, although the most frequently studied, is not the most common headache disorder. Other types of headache, such as the more prevalent tension type headache and sub-types of the more disabling chronic daily headache, have received less attention [1,3]. Globally, the prevalence of the adult population with active headache disorders are 46% for headache in general, 11% for migraine, 42% for tension-type headache and 3% for chronic daily headache [4]. Studies in Europe and United States have shown that migraine affects 6-8% of men and 15-18% of women [5,6]. A similar pattern probably exists in Central America. In Puerto Rico, for example, 6% of men and 17% of women have been found to have migraine [7]. In South America, prevalence appears only slightly lower [8] A survey in Turkey suggested even greater prevalence 9% in men and 29% in women [9]. Migraine appears less prevalent, but still common, elsewhere in Asia (around 8%) and in Africa (3-7%) in few community based studies [1]. In developed countries, tension type headache alone affects two thirds of adult males and over 80% of females [10].

Few population-based studies exist for developing countries where limited funding and large and often rural populations, coupled with the low profile of headache disorders compared with other diseases, prevent the systematic collection of information [1,3,4]. There is a scanty data on the prevalence of primary headaches in sub-Saharan Africa in general and Ethiopia in particular. The prevalence of these headaches is low as compared to Europe and North America [4,11-13]. In 2004, the 1-year prevalence of headache from a door-to-door survey of rural south Tanzania was 23.1% (18.8% males and 26.4% females) [11]. From a 1995 community based study done in rural Ethiopia, the 1-year prevalence of migraine was 3% [12]. In the 2008 urban study done in Akaki textile factory in Ethiopia, lifetime prevalence of all sorts of headaches was 96.1%, 98.6% in females versus 95.9% in males, One-year prevalence of all types headaches was 73.2%, in females 79.2% compared 69.5% in males, The overall 1-year prevalence of all types of primary headaches was found to be 16.4% [13].

Particularly in Ethiopia few studies exists that address this global disabling disorder and there is no urban community study. So in this study we have tried to determine the magnitude of primary headache disorders and its impact. We investigated the urban migraine, tension type headache and cluster headache prevalence and burden using the second edition of the international headache diagnostic criteria (ICHD-II, 2004) [14] for the first time in Addis Ababa. Therefore the objective is to determine one year prevalence and burden of primary headaches and forward recommendations among Addis Ketema sub city, Addis Ababa, Ethiopia.

## Methods

### Study design and population

Study population includes adult population aged 15 and above living in Addis Ketema sub city Kebele 16/17/18 (local smallest administrative unit), which is one of urban sub cities in the city of Addis Ababa, Ethiopia.

Addis Ababa is the capital city of Ethiopia and the African Union and is often called the “African Capital” due to its historical, diplomatic and political significance for the continent. Located in the foothills of the Entoto Mountains and standing 2,355 meters above sea level, it is the third highest capital in the world. It is located in the geographic center of the country. With a population of 2,738,248 as of 2007 census, Addis Ababa is the world’s largest city that is in a landlocked country. Addis Ababa has 10 sub-cities and 99 kebeles (urban dwellers associations), the smallest local administrative units. Addis Ketema sub city is located at the center of Addis Ababa. The sub city is the seat of Merkato the biggest market place of East Africa. The sub city has a total population of 255092, with an area of 8.98 Km^2^ and population density of 25560 people per Km^2^. It has 10 Kebeles and the population of the Kebele 16/17/18 is 1743 people [15].

This locality was chosen by convenience because of restricted financial resource and limited man power to study a large area. Community based cross-sectional design was used to determine prevalence, and magnitude of primary headache disorders.

### Sampling method

The sample size was calculated using the single population proportion formula with the following assumptions: expected prevalence of primary headaches was 16.4% [13] with desired precision of 5% at 95% confidence level and non-response rate of 10%. The sample size was calculated to be 231 and all participated in the study.

### Sampling procedure

The Kebele has 1743 people and 400 households with house numbers. Since our study included only 15 years and above, we calculated the expected number of subjects based on the estimates of the 2007 national census of Ethiopia [15]. Each household contains in average 4.1 members and those aged 15 and above account for 55.1%. Therefore, from a single household we get 4.1 × 55.1/100 = 2.25 people who are age 15 and above. In order to get the sample size of 231, about 102 households were required.

In order to determine sampling interval, the number of houses in the Kebele was divided by the required number of households that gave as the sampling interval i.e., 400/102 ≃ 4, hence every 4th house was taken after selecting the starting point by simple lottery method. To select the households, systematic random sampling was used and the first household was identified through a lottery method.

### Data collection

Data collection was done from March 1/2011 to March 20/2011 using modified previously used headache questionnaires [12,13]. The questionnaires were prepared both in English and Amharic (the national language). They had two parts, the first part includes demographic and personal aspects, and the second part includes specific details of headache. Training was given for two nurse health extension workers on each part of questionnaires. People with positive history of headache were interviewed by the investigators trained in neurology and headache medicine; this was to exclude secondary causes of headache. The diagnosis of each type of primary headache was done by using international Headache Society Diagnostic Criteria (IHS criteria). Those that had secondary headache disorders according to IHS criteria were excluded from the study [15].

### Data analysis

The collected data was processed with a computer with SPSS 11.0 and Epi Enfo version 3.4.1 software packages. Chi-square, Odds ratio, P value <0.05, Fisher exact test and 95% confidence intervals were used for statistical significance.

### Ethical clearance

The research proposal was reviewed and approved by Institutional Review Board (IRB) of the Faculty of Medicine, Addis Ababa University. Informed consent was obtained from the study participants. Since the purpose of the study was explained for each subject those who had headache were informed of the possible solution for their problem and confidentiality was kept. Finally feedback was given to the community under study.

## Result

The total number of study population was 231 and all responded to the questionnaires. Among them 119 (51.5%) were males and 112(48.5%) were females. The age ranged from 16 to 82 with mean of 34.14 ± 14.8 and 79(34.2%) where under 25 years. Of total study population, 107(46.3%) were married, 114(49.4%) were single and 10(4.3%) were divorced. Majority of study population is in secondary school level, 99(42.9%), followed by tertiary level education in 63 (27.3%), elementary school level in 46 (19.9%) and illiterates, those can’t read and write in 23(10.0%) (Table 1).

**Table 1:** Age, educational and marital status by sex distribution of study population, Addis Ketema sub city, Addis Abeba, 2011

**Age category & educational & marital status**	**Female**		**Male**		**Total**	
	**No**	**%**	**No**	**%**	**No**	**%**
15–24	33	29.5	46	38.7	79	34.2
25–34	32	28.5	33	27.7	65	28.1
35–44	18	16.1	16	13.4	34	14.7
45–54	13	11.6	11	9.2	24	10.4
55–64	10	8.9	9	7.6	19	8.2
65 and above	6	5.4	4	3.4	10	4.3
Illiterate	17	15.2	6	5.0	23	10.0
Elementary school	29	25.9	17	14.3	46	19.9
Secondary school	40	35.7	59	49.6	99	42.9
Tertiary	26	23.2	37	31.1	63	27.3
Married	62	53.4	45	57.8	107	46.3
Single	45	40.2	69	58.0	114	49.4
Divorced	5	4.5	5	4.2	10	4.3
Total	112	48.5	119	51.5	231	100.0

About 156 people had headache in the last one year. Out of them 106 (45.9%) were found to have secondary headaches. According to international headache society diagnostic criteria, the overall one year prevalence of all sort of primary headaches was 21.6% (50/231). Out of these 50 cases, 26 (52%) with primary headache disorders were males making a prevalence of 21.8% and in females, the prevalence was 24(21.4%) (OR = 0.98, 95% confidence interval = 0.52-1.83 P = 0.93) (Table 2).

**Table 2:** Prevalence of headache by sex in study population Addis Ketema sub city, Addis Abeba, 2011

**Category**	**Female**		**Male**		**Total**		**OR**	**95% confidence interval**	**P-value**
	**No**	**%**	**No**	**%**	**No**	**%**			
Life time headache	95	84.8	97	81.5	192	83.1	1.27	0.63–2.54	0.62
One-year headache	82	73.2	74	62.2	156	67.5	1.16	0.95–2.90	0.099
**All Primary headache**	**24**	**21.4**	**26**	**21.8**	**50**	**21.6**	**0.98**	**0.52–1.83**	**0.93**
Migraine without aura	6	5.4	9	7.6	15	6.5	0.69	0.24–2.01	0.68
Migraine with aura	5	4.5	1	0.8	6	2.6	5.51	0.63–47.95	0.19
Probable Migraine	1	0.9	1	0.8	2	0.9			
**Subtotal (migraine)**	**12**	**10.8**	**11**	**9.2**	**23**	**10.0**	**0.97**	**0.41–2.30**	**0.88**
Infrequent episodic tension type	2	1.8	3	2.5	5	2.2	0.70	0.12–4.29	0.95
Frequent episodic tension type	8	7.1	11	9.2	19	8.2	0.76	0.29–1.95	0.73
**Subtotal Tension type**	**10**	**8.9**	**14**	**11.9**	**24**	**10.4**	**0.74**	**0.31–1.73**	**0.62**
Cluster headache	0	0.0	3	2.5	3	1.3			
**Total population**	**112**	**48.5**	**119**	**51.5**	**231**	**100.0**			

Age specific prevalence of primary headache disorders was higher in age group 15-24 which 26/79 (32.9%). This was significantly higher in females 48.5% than in males 21.7% (OR = 3.4, 95% confidence interval = 1.27-9.01, P = 0.024) and 47/50 (94%) of all primary headaches were found in the productive age group of 16 to 54 years (Table 3).

**Table 3:** Prevalence of primary headaches by age category Addis Ketema, Addis Abeba, 2011

**Age group**	**Study population No**	**Tension headache**	**Migraine headache**	**All primary headaches**			**OR Or Fisher exact**	**95% confidence interval**	**P-value**
		**No(%)**	**No%**	**Female No(%)**	**Male No(%)**	**Total No(%)**			
15–24	79	10(12.7)	16(20.25)	16(48.5)	10(21.7)	26(32.9)	3.4	1.27–9.01	0.024
25–34	65	5(7.7)	4(6.2)	4(12.5)	5(15.2)	9(13.8)	F = 0.52	0.14–4.17	
35–44	34	3(8.8)	2(5.9)	1(5.6)	4(25.5)	5(14.7)	F = 0.13	0.003–2.182	
45–54	24	6(25.0)	1(4.2)	2(15.4)	5(45.5)	7(29.2)	F = 0.12	0.02–1.97	
55–64	19	0	0	1(10.0)	2(22.2)	3(15.2)	F = 0.46	0.006–9.392	
≥65	10	0	0	0	0	0			
**Total**	**231**	**24(10.4)**	**23(10.0)**	**24(21.4)**	**26(21.6)**	**50 (21.6)**	**0.98**	**0.52–1.83**	**0.93**

The prevalence rate of migraine headache was found to be 23/231 (10%). The prevalence of migraine headache in males was 11/119 (9.2%) while it was 12/112 (10.8%) in females (OR = 0.97, 95% confidence interval = 0.41-2.30, P = 0.88). The prevalence of migraine without aura was 15/231 (6.5%) making15/23 (65.2%) of all migraine patients. This was 9(7.6%) in males and 6(5.4%) in females (OR = 0.69, 95% confidence interval = 0.24-2.01. P = 0.68). The prevalence of migraine with aura was 6(2.6%). This was 5 (4.5%) in females constituting 83% of migraine with aura cases. The prevalence of probable migraine was found to be 0.9% (Table 2).

The prevalence of tension type of headache was 24/231 (10.4%). This was 14/119 (11.9%) in males and 10/112 (8.9%) in females. (OR = 0.74, 95% confidence interval = 0.31-1.73, P = 0.62). Frequent tension type headache was 19/231 (8.2%). This was 11/119 (9.2%) in male and 8/112 (7.1%) in females (OR = 0.76.95% confidence interval = 0.29-1.95, P = 0.73), where as prevalence of infrequent tension type headache was 5/231 (2.2%) which was 2/112 (1.8%) in females and 3/119 (2.5%) in males. Cluster headache was found in three cases (1.3%) exclusively in males (Table 2).

Table 4 shows prevalence of headache versus marital status and missing of days of work, school and social activities. The single and divorced persons showed marginally significant higher proportion of headache prevalence as compared to married people (*X*^2^ =5.31, P = 0.07).

**Table 4:** Primary headache versus marital status missing of working, social or school days, Addis Ketema subcity, Addis Abeba, 2011

**Category of headache**	**Marital status**							***X***^**2**^	**df**	**P value**
	**Married**		**Single**		**Divorced**		**Total**			
	**No**	**%**	**No**	**%**	**No**	**%**	**No**			
**All Primary headache**	**16**	**15.0**	**31**	**27.2**	**3**	**30.0**	**50**	**5.31**	**2**	**0.070**
**Migraine**	**7**	**6.5**	**14**	**12.3**	**2**	**20.0**	**23**	**1.42**	**2**	**0.491**
**Tension type headache**	**9**	**8.4**	**14**	**12.3**	**1**	**10.0**	**24**	**0.005**	**2**	**0.997**
	Missing days									
	No			%						
Migraine	18			78.3				20.4	1	0.000
Migraine without aura	10			66.7				6.8	1	0.009
Migraine with aura	5			83.3				6.2	1	0.013
Tension type	16			66.7				11.4	1	0.001
Infrequent tension type	2			40.0						
Frequent tension type	14			73.7				13.2	1	0.000
All primary headaches	34			68.0				29.4	1	0.000

All primary headache disorders were associated with significant missing of working, school, or social activities. All types of primary headaches lost days in 68.0% (*X*^2^ = 29.4, P <0.005). Migraineurs 18/23 (78.3%) missed days (*X*^2^ = 20.4, P <0.001) the worst being in migraine with aura 83.3% (*X*^2^ = 6.2, P <0.05) followed by migraine without aura in 66.7% (*X*^2^ = 6.8, P < 0.01). Tension type headache lost days in 66.7% (*X*^2^ = 11.4, P <0.005). Frequent episodic tension type headache cases missed days in 73.7% (*X*^2^ = 13.2, P <0.005).

The overall health seeking practice to health facilities is less than 8/50 (8.0%) in all primary headache sufferers. About 92.0% (42/50) did not visit health institutions at all during their acute headache attacks. Sixty six percent (28/50) did not use modern medicine any time to abort headache attacks and none were using preventive therapy. And yet from 17% to 52% of the different primary headache category cases took treatment for the attacks of headache signifying self over the counter medication or other options. The most frequently used medicine was paracetamole.

## Discussion

There was a slight male preponderance of the study population. More than 34% were under 25 years of age with mean age of 34 years and about 83% were in the age group between 16 to 54 years of productive age. This is similar to the 1995 and 2008 Ethiopian and 2009 Pakistan studies in which about 80% of the patients were below 55 years of age [12,13,16].

The overall 1-year prevalence of primary headache disorders in this study was 22%. This is higher than studies done previously in Ethiopia [12,13]. This is similar to the prevalence for Africa which is 21% and the 2012 study from China of 24% [17], but much lower than that of the global estimate which was 46%[4]. The highest age specific prevalence of all primary headaches in this study 34% in the age group 15-24 was in the young population. The prevalence in this study and that of the different countries studied previously are compared in Table 5.

**Table 5:** The prevalence primary headaches in the current study compared to previous publications in different countries

**Author/country**	**Journal**	**Year of publication**	**Primary headache%**	**Migraine%**	**Tension type headache%**	**Chronic tension headache/CDH%**	**Cluster headache%**
**In this study**			**21.6**	**10.0**	**10.4**	**0.0**	**1.3**
Takele Ethiopia	J headache pain	2008	16.4	6.2	9.8	1.6	0.45
Yu China	Headache	2012	23.8	9.3	10.8	1.0	NA
Teklehaimanot Ethiopa	cephalalgia	1995	NA	3.0	NA	1.7	0.03
Queiroz Brazil	cephalalgia	2005	NA	22.1	22.9	6.4	NA
Stovner Global	cephalalgia	2007	46.0	11.0	42.0	3.0	NA
Cheung Hong Kong	Headache	2000	37.1	4.7	26.9	1.0	NA
Ertas Turkey	J headache pain	2012	NA	28.8	14.5	0.1	NA
Yoon Germany	J headache pain	2012	NA	16.6	12.5	0.5	NA
Katsarava Georgia	J headache pain	2007	NA	8.6	20.4	5.4	NA
Katsarava Georgia	Neurology	2009	NA	15.6	37,3	3.3	NA

Less well recognized is the toll of chronic daily headache: up to one adult in 20 has headache on more days than not [1,18,19]. Furthermore, several (though not all) follow-up studies in developed countries suggest that headache prevalence and burden are increasing [20]. However chronic tension type of headache is absent in this study which is may be due to the small sample size.

One year prevalence of migraine headache in this study was 10% which is closer to global prevalence of migraine headache that is 11% [1] and it is found to be higher than previous studies done in Ethiopia; one was rural community based study which showed 3% [12] and in the other study done in Akaki textile factory which was 6.2% [14].

The 10% prevalence of migraine in this study is similar to the 2012 Chinese study 9.3% [17] but lower than the study done in that of 2007 Georgia’s prevalence of migraine headache which was 22% [21] and the 2012 nationwide study of Turkey, in which the 1-year prevalence of definite migraine was estimated to be 16% and all types of migraine was 29% [22]. In the 2012 headache consortium study of Germany, the prevalence of all types of migraine was about 18% [23]. It is well known the prevalence in developed nations of America and Europe is much higher than that of Africa and Asia. Prevalence Estimates in those studies that did not use IHS criteria and done in clinics or hospitals may not show the true community images.

Migraine without aura in this study takes the higher proportion of migraine headache which is 65% and that of migraine with aura was 26% of migraine headache in general; this is true in most studies [1,4,12,13,23-25].

The one year overall prevalence of tension type of headache in this study was 10.4% which is similar to the 2012 Chinese study of 10.8% [17] and closer to the study done in Akaki textile factory in Ethiopia [13], the German Consortium study of 13% [23] and the 2012 Turkey study which was about 15% [22] but lower than that of studies done in Japan (20%) [4], Georgia (20%) [21] And much lower than 2007 global estimate which was 42% [4]. This prevalence rate is much higher than WHO report of tension type of headache for Africa which is 1.7% [1]. This prevalence is much lower than other studies done elsewhere [24,26].

The prevalence of cluster headache is found to be 1.3% exclusively in males in this study. This is also true in other studies (1, 4 12, 13, and 25) and is a disease much of the males in the world.

The prevalence of primary headaches seems to be higher in the urban setting like in this study as compared to the rural communities in Ethiopia The reasons for such differences might be: 1) the rural people have greater tolerance to pain. The physical labor, hard work, exercise and normal or low body mass index might contribute to the tolerance and lesser prevalence of headache in the rural people. This is exemplified by the 2012 Chinese study which showed that the body mass index of more than 30 to be associated with higher prevalence of migraine [27] and the 2011 Danish study in which a decreased risk of migraine was significantly associated with heavy exercise [28] 2) Primary headaches, even if present and recurrent, are often trivialized, because of more demanding and basic problems of organic causes of headache and infections, as well as discrimination of children and women in the rural people. 3) Rural sufferers of headache are from the lowest socioeconomic segment and are less educated and hence they lack the awareness. 4) The prevalent magico-religious perception of diseases and opting for traditional healing in rural communities may hinder the reporting of headaches [13,25].

In this study significant portion of primary headache cases, particularly migraine and tension type of headache, were missing working, school and social days, and house hold activities during their headache attack. This is 78% of migraine and 67% of tension type headache cases. Repeated headache attacks, and often the constant fear of the next, damage family life, social life and employment [29]. For example, social activity and work capacity are reduced in almost all people with migraine and in 60% of those with tension-type headache. Headache often results in the cancellation of social activities while, at work, people who suffer frequent attacks are likely to be seen as unreliable — which they may be — or unable to cope. This can reduce the likelihood of promotion and undermine career and financial prospects [29].

While people actually affected by headache disorders bear much of their burden, they do not carry it all: employers, fellow workers, family and friends may be required to take on work and duties abandoned by headache sufferers. Because headache disorders are most troublesome in the productive years (late teens to 60 years of age), estimates of their financial cost to society are massive — principally from lost working hours and reduced productivity because of impaired working effectiveness and absentees during attacks[28]. In the United Kingdom, for example, some 25 million working or school days are lost every year because of migraine alone [6].

Therefore, while headache rarely signals serious underlying illness, its public health importance lies in its causal association with these personal and societal burdens of pain, disability, damaged quality of life and financial cost. Not surprisingly, headache is high among causes of consulting both general practitioners and neurologists [18,19]. One in six patients aged 16–65 years in a large general practice in the United Kingdom consulted at least once because of headache over an observed period of five years, and almost 10% of them were referred to secondary care [30]. A survey of neurologists found that up to a third of all their patients consulted because of headache — more than for any other single complaint [31].

In the 2008 Ethiopian study at a textile mill factory in Addis Ababa, more than 60% with migraine and above 20% with tension headache missed working days and almost all with migraine as well as more than half with tension headache have a reduced work capacity during the acute attacks of headache [13]. The quality of life is the worst of all the compromises for the individual cases. Furthermore only 8% of cases with primary headache disorder practiced health seeking behavior in this study. Headache is a big problem also in less affluent countries where modern pharmacologic treatment is, and probably for the coming years will be, less accessible [23,32,33]. Pain, suffering and disability caused by headache disorders impose substantial burdens, at both personal and societal levels [17,34]. Although the burden of headache is large on all continents, headache prevalence and burden are poorly described in large and populous regions like Africa and Asia [4,25].

In most African countries in general and in Ethiopia in particular seeking medical care is delayed or unavailable. This is complicated by under diagnosing or misdiagnosing and unrecognizing of primary headaches. Moreover there are barriers to the effective care and diagnosis of these disorders in the world in general and in Africa and Ethiopia in particular. Lack of knowledge among health care providers is the principal clinical barrier. This starts in the medical schools, where the curricula are deficient and where there is limited or no teaching on the subject. This is verified by a 2008 the UK study where 70% of headaches were not given a diagnostic label, by general practitioners (GPs). It is suggested that GPs’ difficulty in diagnosing headache presentations contributes to the high level of morbidity and unmet need in this disease in such a developed nation leave alone in Africa [1,35].

Almost all physicians except few neurologists or headache specialists in Ethiopia did not know the specific therapy to abort the acute episodic attacks and the preventive therapy that reduce the frequency and severity of the suffering and disability. This is exemplified by that there was no a single case on specific therapy or preventive therapy in this study. Trivialization of headache and poor awareness extended largely to the public compounded by gender and children discrimination is the culprit of the social barriers. Moreover many of the sufferers are unaware that effective treatment and preventive therapy exists. Political and economic barriers account a lot especially in developing nations like Ethiopia, where there are no specific therapy drugs like tryptans or ergot derivatives like cafergot. Many governmental and nongovernmental stake holders do not recognize that the direct costs of treating headache are smaller in comparison with the huge indirect-cost savings that might be made from reducing lost working days, school days, social activities, etc [1,13,17,20,25,33].

Migraineurs with aura had the greatest level of distress in this study, followed by migraine without aura and tension-type headache, which is a similar finding to the 30 years Swiss prospective study in 2011 [36].

## Conclusion

Primary headache disorders are common in this community based sample study and the burden of them is intense and massive. Health seeking behavior of community is inadequate. There are no drugs for specific therapy of primary headaches in Ethiopia currently. So we recommend emphasis and public awareness on primary headache disorders through health education and public media. Specific therapy drugs like the tryptans or ergot derivatives should be available in the country. We also recommend a national large community based study comparing the urban with the rural population.

### Limitation of the study

The small sample and the fact that we did not use directly MIDAS (Migraine disability assessment score) are the limitations of this study.

## Competing interest

The authors declare that they have no competing interests.

## Authors’ contribution

GM-design, questionnaire setting compiling analysis and write up, SA: data collection, compiling and write up. Both authors read and approved the final manuscript.
